# How are pluripotent cells captured in culture?

**DOI:** 10.1007/s12522-014-0199-8

**Published:** 2014-12-03

**Authors:** Masaki Kinoshita

**Affiliations:** ^1^ Wellcome Trust‐Medical Research Council Stem Cell Institute University of Cambridge Tennis Court Road CB2 1QR Cambridge UK

**Keywords:** EG cells, Epiblast, EpiSC, ES cell, Pluripotency

## Abstract

In mice, three pluripotent stem cell lines have been established from different stage of developing embryo, which are embryonic stem (ES) cell, post‐implantation epiblast stem cell (EpiSC), and embryonic germ (EG) cell. ES cell and EG cell share many common features including factor requirement, colony morphology, and gene expression pattern. On the other hand, EpiSC needs different external signal inputs, exhibits flattened colony morphology, and a different set of gene expression patterns. In addition, the germ line competency of EpiSCs is still unclear. To distinguish the differences between them, they are defined by the words “naïve” and “primed” pluripotent cells, respectively. This article introduces how pluripotent stem cell lines are established in culture, and how much those cells in vitro are similar or relevant to their in vivo origin and the knowledge about transcription factors to support this state.

## Introduction

Pluripotency is the word that represents the cell state that gives rise to all three germ layers: ectoderm, mesoderm, and endoderm. Pluripotent cells only exist at the early starting point of our lifetime. Importantly, this pluripotent cell state is a transient one, which can be observed in mammalian development. We are now capable of capturing some of these pluripotent cells in culture. According to the recent progress of the imaging tools as well as embryo culture, we can monitor peri‐implantation development in vitro relatively easily and also genetics allows us to examine the gene function in a stage‐ or tissue‐specific manner. Thanks to these techniques, our understanding about how the mammalian embryo develops from fertilized egg is deepened, as is our knowledge about pluripotent stem cell in culture, especially for mice. In this article, I describe pluripotency in vivo and in vitro and their relationship together with the external signals and gene functions which support their status.

## Pluripotent cells in mouse development

### How totipotent cells become pluripotent cells

**Figure 1:**
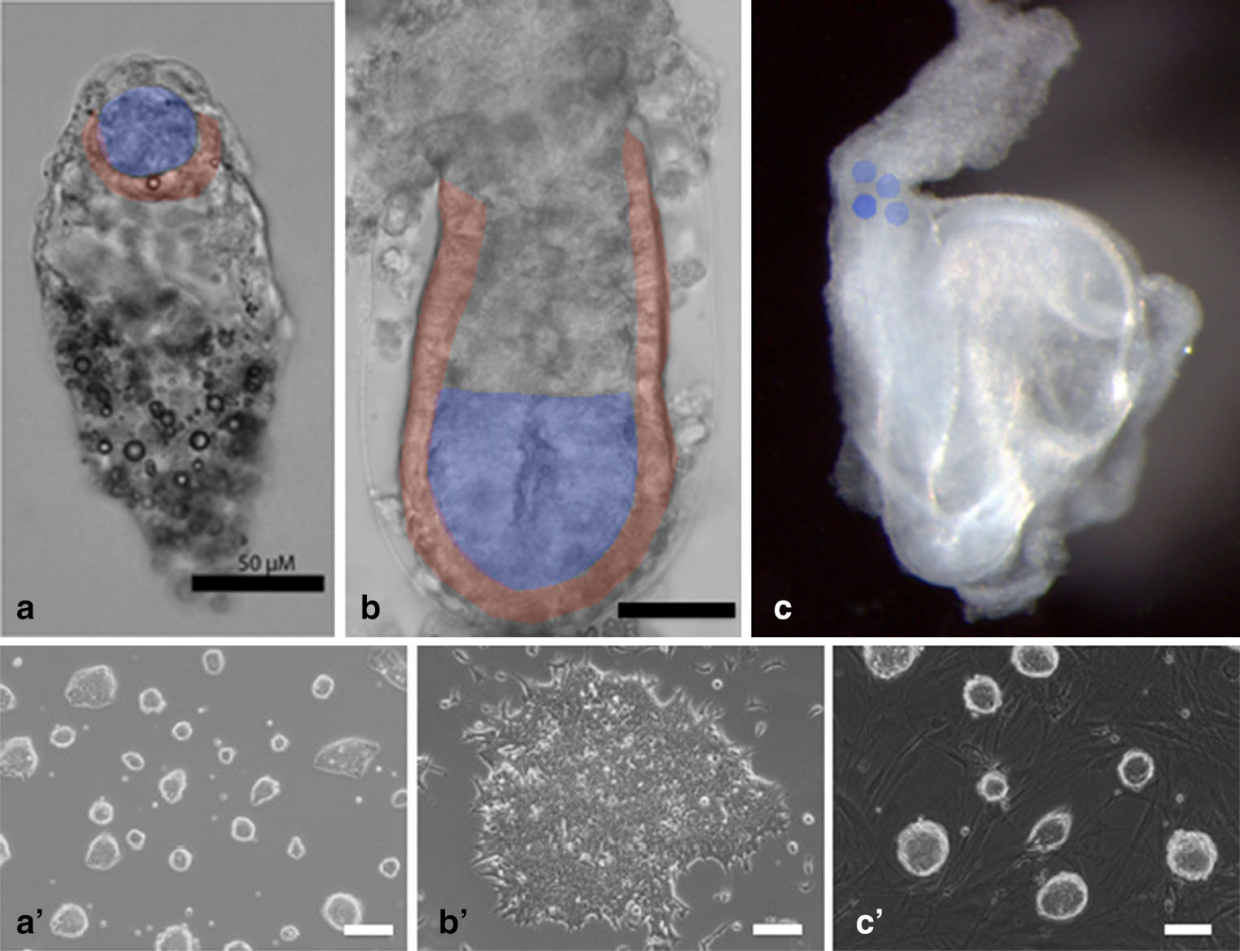
Pluripotent cells in culture and their origin in embryos **a** E4.5 mouse embryo. **a’** Mouse embryonic stem cell cultured in 2i and LIF on a gelatin‐coated plate. **b** E5.5 mouse embryo. **b’** Mouse EpiSC cultured in activin and bFGF on fibronectin‐coated plate. **c** E8.5 mouse embryo. **c’** Mouse EG cells cultured in 2i and LIF with feeder cells. Cells in *blue* in (**a**, **b**) show the pluripotent epiblast and in **c** show the location of PGC cells at this stage. *Red* cells in (**a**, **b**) are extra‐embryonic endoderm cells. *Scale bar* in **a**, **b** is 50 µm and **a’**–**c’** is 100 µm

The fertilized egg of mice is a totipotent cell in definition because this unique cell can become every type of cell, including extraembryonic tissues such as placenta or yolk sac. After fertilization, the cell divides without increasing the total embryo size, a process called cleavage, and cells of the embryo around this stage look identical until the eight‐cell stage. At the eight‐cell stage, compaction occurs, and cell–cell interaction causes polarization. The cells located on the outer surface of the embryo around the 8–16‐cell stage embryo become trophectoderm, which contributes to the future placenta [1, 2], and this is the first extraembryonic lineage determined in embryonic development. As the embryo develops, inside cells locate on the inside wall of the trophectoderm layer as an aggregate called inner cell mass (ICM). ICM at this stage consists of a mixed population of future epiblast cells (which contribute to the embryo) and extraembryonic endoderm cells, which is the second lineage segregated from the embryonic lineage [3]. Just before implantation, the cells at the surface of the blastocoel commit to their fate of primitive endoderm (PrEn) as a morphologically visible single layer (Fig. 1a in red color) [3]. The mechanism for cell sorting is not well understood, but there is a tendency for cells destined to become primitive endoderm travel through the ICM toward the blastocoel in an actin‐dependent manner [4, 5]. Epiblast cells after the PrEn cells segregated (Fig. 1a, blue color) are the pluripotent cells because all of the somatic cells are derived from these cells.

### Epiblast cells after implantation

After implantation, apolarized ICM cells continue to proliferate and line up as one sheet of epithelial cells which have an apical‐basal polarity in a cup‐shaped structure called an egg cylinder in rodents, disc in other mammals. The polarized epiblast cells are attached to the basement membrane produced by surrounding visceral endoderm cells (Fig. 1b). Recent study suggests that apical constriction of basement membrane‐anchored ICM cells causes cavity formation at the apical surface of ICM cells [6]. Another study from embryoid body‐based cavity formation analysis [7, 8] shows that cells that are not incorporated into the epithelial layer undergo apoptosis. These mechanisms both contribute to make proamniotic cavity.

Epiblast cells collected from embryonic day (E)6 and E7 embryo do not colonize the embryo when injected into the blastocyst stage [9], but these cells were shown to make all three germ layer derivatives when ectopically introduced into another host animals [10, 11]. The cells constituting postimplantation epiblast have been shown to contribute efficiently to PGCs in vitro when cultured in high doses of bone morphogenetic protein (BMP). These studies show that post‐implantation epiblast cells still harbor pluripotency that can give rise to any types of the cells, including germ cells [12].

### Unipotent cell converts to pluripotent cell in vivo

Another type of pluripotency‐related cell observed in the developing embryo is the germ cell. Germ cells are unipotent cells that normally give rise to sperm or egg. In developing mice, primordial germ cells (PGCs) firstly emerge around the pre‐gastrulation stage as a few Blimp1 expressing alkaline phosphatase (AP)‐positive cells in the posterior proximal epiblast [13, 14]. These cells proliferate and repress the somatic gene programs during gastrulation [15]. They form AP‐positive cell clusters at the bottom of the allantois. Then PGCs migrate along the hindgut and colonize the aorta‐gonad‐mesonephros (AGM) region until around E12.5 to become mature germ cells. Though PGC itself in vivo is not pluripotent but uni‐potent, these PGCs are the origin of embryonal carcinoma (EC), which is sometimes observed in 129 mice strains [16]. EC tumors contain three germ layer derivatives, so PGC is not normally pluripotent, but rarely, it converts to pluripotent cell state in vivo.

## Pluripotency in culture

### Pluripotent cells from peri‐implantation‐stage epiblast

As introduced above, there are transient pluripotent cell states during development, and nowadays we can establish pluripotent stem cell lines from these different stages of the developing embryo. Mouse embryonic stem (ES) cells (Fig. 1a’) are established from pre‐implantation stage ICMs (E3.5–E4.5), post‐implantation epiblast stem cells (EpiSCs) (Fig. 1b’) are established from peri‐gastrulating embryo (E5.5–E8.0), and we also can establish pluripotent stem cells in vitro from PGCs [from E8.5 (Fig. 1c) to E12.5], called embryonic germ (EG) cells (Fig. 1c’) [17, 18] (Table 1). Interestingly, ES and EG cells have different origins in terms of developmental stage, but they share common features including culture condition, growth factor requirement and chimera formation ability.

**Table 1:**
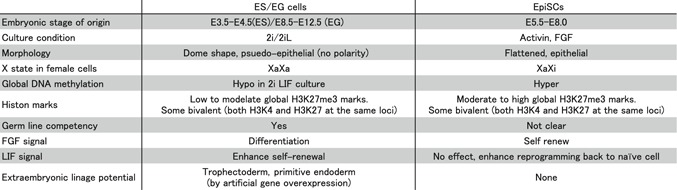
Features of naïve and primed cells

Historically, in vivo pluripotency was first shown in EC cells [19]. When these cells are ectopically transplanted into a recipient animal, they give rise to tumors that consist of three germ layer‐derived cells. EC cells were then shown to have the ability to contribute to host embryo development to make a chimeric animal when injected into blastocyst‐stage embryos [20], but only a few EC cell lines have this ability. Reliable contribution to chimera is one of the special features of mouse ES cells, different from EC cells, focused on in this article.

mES cells were first established from the ICM of the blastocyst‐stage embryo in 1981 [21, 22]. mES cells injected into a host embryo can contribute to every somatic lineage including germ cells in accordance with the host embryo development. Contrary to the features that EC cells are different from line to line, ES cells are more similar to each other in terms of morphology, culture condition, and ability for making chimeric animal. Since then, researchers are in pursuit to understand why these cells can be kept undifferentiated in vitro. ES cells are derived from the early embryo, so can be used for understanding early mammalian development as an in vitro model, and also human ES cells can be expected to be used for clinical applications in so‐called “regenerative medicine.” Another useful role of ES cells is making transgenic animals. Because ES cells can contribute to every cell type including germ cells, once the transgenic ES cells are established by homologous recombination of the gene of interest, mating the chimeric animal to a wild type allows establishment of new mouse lines that can be used for gene function analysis.

### Pluripotent cells from post‐implantation epiblast

EpiSCs has been reported in 2007 from mouse post‐implantation‐stage embryo [23, 24] and rat post‐implantation‐stage embryo [23]. This pluripotent cell line is established from post‐implantation‐stage epiblast, so it is called post‐implantation epiblast stem cell (EpiSC) to distinguish from ES cells established from pre‐implantation‐stage epiblast. EpiSC lines have some similarities to human ES cells that are established from human blastocysts [25]. For example, growth factor requirements for these cells are basic fibroblast growth factor (bFgf) and activin instead of leukemia inhibitory factor (LIF). Their morphology is a flat two‐dimensional colony, but mES cells are dome‐shaped and three‐dimensional. Pluripotency of these cells is shown by teratoma forming ability when ectopically transplanted into immune‐deficient host animals, but when injected into blastocysts, mEpiSCs very rarely contribute to a chimeric animal [23]. Recently, E‐cadherin overexpressing EpiSC was reported to contribute to chimeras by blastocyst injection, but the underlying mechanism is still unknown [26], and no germ line contribution of these cells was observed. Another group injected EpiSC into ex vivo cultured egg cylinder‐stage embryos of the equivalent developmental stage to EpiSC origin. They reported that injected EpiSC into E7.5 embryo incorporate into the host and contribute to chimeric tissue including AP‐positive, putative germ cells [27]. In human ES cell differentiation culture, there are some reports that hES cells differentiate into VASA‐positive pre‐meiotic germ cells in three‐dimensional culture, though it is still controversial whether these types of stem cells have the potency to become germ cells [28, 29, 30].

Another similarity is that both human ESC and mouse EpiSC are fragile when dissociated at the single cell level; they activate the pathway to apoptotic cell death triggered by blebbing. When Rho‐associated coiled‐coil containing protein kinase (ROCK) inhibitor is added to block this, cell death can be avoidable [31, 32].

### Pluripotent cells from PGC

EG cells were first established from PGCs of E8.5 and E12.5 in the presence of stem cell factor (SCF), LIF, and bFGF [17], and it has been confirmed that they are germ line‐competent cell lines [33, 34]. As described above, PGC in vivo is a uni‐potent cell that only makes sperm or egg, so the conversion of PGC to EG cell is an in vitro reprogramming process. Once they become EG cells, they are very similar to ES cells in every aspect. For example, they can grow in serum and LIF or 2i condition (a recently establish culture condition for ES cells containing two kinase inhibitors [35], described later in this article), and their gene expression profiles and DNA methylation status are similar to those of ES cells [36].

### Naïve and primed pluripotency

Because of obvious differences between established pluripotent stem cells, the pluripotent state of EpiSC is defined as “primed pluripotency” to distinguish from mES and mEG cells’ “naïve pluripotency” [37]. Naïve and primed states have some more, different features (listed in Table 1). One example is that female naïve mES cells have both active X chromosomes (XaXa), but primed female stem cells have only one active chromosome and the other is inactivated (XaXi). This might represent the difference of the developmental stage from which each type of cells is established.

### Reprogrammed pluripotency

In 2006, Takahashi and Yamanaka found a new technology to induce pluripotent stem cells from terminally differentiated somatic cells by the specific combination of transgenes (Oct3/4, Sox2, Klf4, and cMyc) [38]. The finding was striking in showing that the key transcription factor(s) are sufficient to change the fate into totally different types of cells. These reprogrammed cells are named induced pluripotent stem (iPS) cells. When these Yamanaka factors are introduced into mouse embryonic fibroblasts, they form the dome‐shaped colony that can be maintained similar to ES cells and these reprogrammed cells can colonize a host animal when injected into the blastocyst‐stage embryo to make a chimeric mouse. Importantly, germ line competency of the iPS‐derived chimeras were confirmed in 2007 [39, 40, 41]. In 2011, Hayashi et al. [42] succeeded in inducing mouse PGC‐like cells from male ES cells as well as male iPS cells, which can be a functional sperm when introduced into seminiferous tubules of the host male. They also succeeded in making functional female PGC‐like cells from both ES cells and iPS cells [43]. These female PGC‐like cells can become mature oocytes when transplanted into host animals and make offspring by in vitro fertilization.

iPS technology is applied to reprogram human somatic cells as well. This technology is really useful because we can utilize patient‐derived iPS as a disease model to uncover the mechanism of disease development and drug discovery, as well as a tool to understand human development and a potential application for future cell or tissue replacement therapy directly.

## Development of culture conditions for mES cells

### Culture condition towards serum free

ES cells had been cultured on mouse embryonic fibroblast cells as a feeder of essential factor(s) for maintenance with strictly tested and selected serum as a source of growth factor supplement. In 1988, Smith et al. and Williams et al. [44, 45] reported that one cytokine, LIF, could support ES cell self‐renewal without feeder cells, and showed that LIF is the essential factor provided by feeder cells. Smith et al. [46] further developed serum‐free culture conditions with BMP4 and LIF. mES cells are known to be prone to differentiate into the neural cell lineage in serum‐free media, and what they showed is that inhibition of neural induction by BMP4 in addition to activation of STAT3 by LIF signaling is sufficient to block differentiation and maintain pluripotent self‐renewal. Serum can be replaced by chemically defined supplement, knockout serum replacement (KSR from Life Technologies), and ES cells can be cultured without feeder cells in the presence of LIF, but it is not sufficient to propagate from single cell in this condition. Ogawa et al. [47] established adrenocorticotropic hormone (ACTH), KSR and LIF conditions to grow at clonal density.

In 2008, Qi‐Long Ying et al. [35] established a novel serum‐free ES cell culture method that contains two kinase inhibitors for GSK3 (CHIR99021) and Mek inhibition (PD0325901) in the basal medium, so now it is simply called 2i. This 2i condition firstly enabled us to culture ES cells without LIF or its downstream signal transducer, Stat3. In addition to its lack of necessity for LIF, this 2i condition allows us to establish NOD mouse ES cell lines [48, 49] and rat ES cell lines [50, 51], which were impossible to establish before the discovery. In addition to rat ES cells, the 2i condition allows us to establish rat EG cells at the first time [52]. This condition allows us to culture mES cells in a more homogeneous manner, which is called “ground state” of mES cell.

### ES cells cultured in serum and LIF

It is also well known that undifferentiated ES cells cultured in conventional ES cell culture conditions such as serum and LIF without feeders are very heterogeneous in terms of gene expression and morphology. For example, Oct3/4 (also known as Pou5f1) is widely expressed in serum containing culture conditions, but Rex1 (also known as Zfp42) is not. Toyooka et al. [53] established a GFP knock‐in reporter ES cell line in *Rex1* gene locus and showed that Rex1‐GFP‐positive cells emerged from its negative fraction and vice versa, but Rex1‐negative cells did not contribute to chimeras. From this observation, ES cells are considered to fluctuate between partially differentiated and undifferentiated status under serum and LIF conditions. Genes such as Nanog, Dppa3, Klf4, Tbx3, and Esrrb are also known to fluctuate in this culture condition [54, 55, 56, 57].

## What makes it possible to keep cells undifferentiated in culture?

### LIF signaling

Before the discovery of 2i culture, LIF was the sole molecule known to support self‐renewal of mouse ES cells in the presence of serum‐derived factors, thus its downstream signal and transcription factor network was extensively examined for a few decades. LIF is a cytokine that belongs to the interleukin 6 family and binds to LIF receptor to make a heterodimer with gp130 (also known as Il6st). This dimerization makes Janus Kinase (JAK) phosphorylate gp130 and Stat3. Phosphorylated Stat3 trans‐locates into the nucleus where it works as a transcription factor in ES cells [58]. gp130 is also known to activate Ras‐Mapk signal and PI3‐Akt signal pathways in parallel to Stat3. Firstly, Stat3 was shown to be a sufficient molecule to support LIF‐independent self‐renewal [59]. Matsuda et al. constructed a fusion protein that has a modified ligand binding domain of the estrogen receptor combined at the c‐terminal of Stat3, called Stat3‐ER. The localization of this fusion protein is controlled by the addition of 4‐hydroxytamoxifen (4‐OHT). They reported that the addition of 4‐OHT in media without LIF (this recruits the fusion protein into the nucleus) is sufficient to support self‐renewal. From this observation, the Stat3 pathway is considered as the main pathway activated by LIF.

### Downstream of Stat3 target

By over‐expressing the gene of interest in ES cells, we can check their ability to support LIF‐independent self‐renewal. Like forced nuclear localization of Stat3 supporting LIF‐independent self‐renewal [59], Nanog, Esrrb, Tbx3, Klf2, Klf4, Klf5, Gbx2, and Tfcp2l1 are also identified to be able to bypass LIF‐Stat3 signaling [56, 60, 61, 62, 63, 64, 65, 66, 67]. This showed that these transcription factors make a gene regulatory network in parallel or downstream of Stat3. In addition to these transcription factors, PI3 kinase and Akt signaling activated by LIF and gp130 were also reported to support self‐renewal of mES cells [68, 69].

### FGF‐Mapk signal

Among the Fgf family molecules, Fgf4 is the main Fgf produced by mES cells. Fgf4 starts to be expressed at around 4–8‐cell‐stage embryo, and continues its expression in the ICM of blastocyst and egg cylinder stages [70]. Genetically inactivated *Fgf4*‐null embryos fail to implant or produce pluripotent ICM outgrowth in vitro, so Fgf4 was considered as a molecule that supports proliferation of pluripotent cells in vivo and in vitro in an autocrine manner [71]. To test this possibility, *Fgf4* null ES cells have been established, and found to have no effect in proliferation or maintenance of the undifferentiated state [72]. Kunath et al. [73] showed in 2007 that Fgf4 is essential for exit from self‐renewal to differentiate. They showed that *Fgf4* null ES cells can differentiate neither into neural nor mesoderm lineages without the addition of Fgf4 into the media. Fgf activates PI3 K in addition to Ras‐Mapk pathways. They also showed that Erk2 is the main downstream molecule that corresponds to this Fgf4 signaling by using *Erk2* KO ES cells.

## Essential genes for keeping the specific gene regulatory network in the mES cell

A number of genes have been identified that are highly or specifically expressed in undifferentiated ES cells compared to somatic cell lines or cancer cell lines, although only a few genes have been reported to play an essential role in the maintenance of undifferentiated mES cells (Table 2). Oct3/4 is one such essential key player in organizing the transcription factor network. On the other hand, Sox2 is expressed in many other cell types and cancer, however it makes heterodimer with Oct3/4 and plays a crucial role in ES cells. These two genes are the original half of the Yamanaka four‐factor cocktail with Klf4 and cMyc, which are sufficient for somatic cell reprogramming [38]. In this section, I introduce some of them on top of Oct3/4 and Sox2.

**Table 2:**
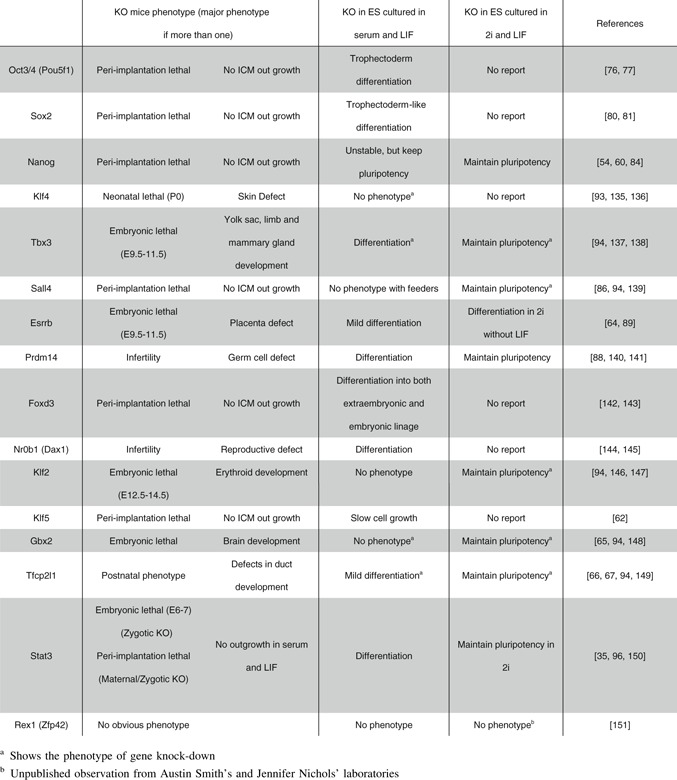
List of gene knockout/knockdown phenotypes in mouse and ES cells

^a^Shows the phenotype of gene knock‐down

^b^Unpublished observation from Austin Smith's and Jennifer Nichols’ laboratories

### Oct3/4

Oct3/4 (also known as Pou5f1) was reported by two groups independently, as Oct3 [74] and Oct4 [75], and so this molecule is called Oct3/4 in this review. Oct3/4 is a homeobox protein, and binds to octamer DNA motif. Oct3/4 is expressed in pre‐implantation‐stage embryos and also supplied as a maternal factor from oocytes. *Oct3/4* knockout embryos fail to establish a pluripotent inner cell mass at the blastocyst stage and die around implantation [76], showing that it is required for early development. Oct3/4 is proven to be essential for maintenance of in vitro pluripotency by conditional knockout in ES cells [77]. ZHBTc4 ES cells do not harbor both endogenous *Pou5f1* gene loci but are maintained undifferentiated by a tetracycline (Tet) inducible *Oct3/4* transgene. Tet addition into the culture media results in a rapid loss of Oct3/4 protein and differentiation into trophectoderm cells through de‐repressing the Cdx2 and Eomesodermin genes [78].

### Sox2

Sox2 is a member of the high‐mobility group of transcription factors that consist of 21 genes. Different from the expression pattern of Oct3/4, which is restricted to pluripotent epiblast and germ cell lineages during early development in mice, Sox2 expression is more broad in the embryo, and it is also expressed in the trophectoderm cell lineage in addition to epiblast [79]. *Sox2* KO embryos can implant but fail to propagate pluripotent epiblast, resulting in lethality before gastrulation [80]. This phenotype is different from Oct3/4. *Sox2* KO ES cells are established by using a similar approach to Oct3/4 [81]. *Sox2* deletion also causes differentiation into trophectoderm‐like cells but interestingly this *Sox2* deletion mutant ES cell phenotype was rescued by forced expression of Oct3/4, indicating that its function overlaps. Actually, it is now also known that Oct3/4 and Sox2 make hetero dimer and co‐bind to the same locus to maintain pluripotency [82, 83]. Deletion of Oct3/4 cause the rapid down regulation of known common target genes [58, 78], but Sox2 deletion needs more time to down‐regulate these genes [81], suggesting that Sox2 function is more supportive in these common target sites.

### Nanog

Another important molecule that is required for the establishment of pluripotent cells is Nanog. Nanog was identified most recently compared to other important classic genes. Nanog, which belongs to a homeobox protein group, was discovered by two independent groups, and named after the mythological Celtic land of ever young, “tir nan og” [60, 84]. Nanog expression starts at the compacted morula stage, and then becomes restricted to the ICM of the blastocyst, but is quickly down‐regulated at around the time of implantation [60]. Nanog is essential for establishment of in vivo pluripotency [84] and also required for in vitro reprogramming of differentiated cells [85], but is not required for maintenance of pluripotency in vitro [54].

### Most of the transcription factors required for in vivo epiblast formation are not required for in vitro pluripotency

Oct3/4 and Sox2 are important molecules for the pluripotency‐associated gene regulatory network, but there are several genes that play important roles in the establishment of in vivo pluripotent epiblast cells but not essential for the maintenance in vitro, such as Nanog. Also, there are several genes that are thought to be important by gene overexpression studies, but KO has no phenotype. These are summarized in Table 2, and some of them are described more in this text. *Sall4* KO mice fail to develop after implantation, although both ICM and TE cells appear intact at the blastocyst stage. *Sall4* null ICM has difficulty in propagating in vitro to establish ES cell lines [86]. To address the roles in the maintenance of gene networks in ESCs, conditional KO was performed using a floxed allele, and from this study, Sall4 was found not to be required for the gene regulatory networks, but for repression of TE differentiation through the direct interaction with histone deacetylase (HDAC) [87].

Prdm14 (PR domain containing 14) was identified as an essential gene for establishment of the germ cell lineage in mice [88]. It is reported in conventional ES cell culture medium containing serum and LIF that *Prdm14* KO ES cells cannot be established, but it is possible by using 2i culture. *Prdm14* KO ES cells have an elevated level of Fgf signaling pathway, so in the presence of Mek inhibitor, *Prdm14* null cells can be maintained. *Prdm14* null cells also have an increased level of DNA methylation at the de novo methylation site by the up‐regulation of DNA methyltransferase (Dnmt) genes. Prdm14 protein physically interacts with Jarid2 and Suz12, members of the Polycomb repressive complex (PRC) 2, suggesting that the function of Prdm14 is to recruit PRC2 complex into the promoter region that is active in primed state epiblast cells including Dnmts.

Esrrb was recently reported as a downstream molecule of LIF signaling [64] and Nanog [63], and *Esrrb* (‐/‐) ES cells can be maintained in conventional culture [64]. However, in 2i culture, *Esrrb* (‐/‐) is essential for maintenance of pluripotency as an important GSK3 inhibition target, so it is considered as an important molecule in the maintenance of naïve pluripotency. From KO study, the *Esrrb* gene is apparently not required for epiblast development [89].

Single KO might be compensating for the other functional overlapping factors. Some of the transcription factors have other closely related family members, which are expressed together. For example, in the developing embryo, double KO of Gata1 and Gata2 [90], Klf2 and Klf4 [91] cause more severe phenotypes in blood cell development than single KO, or another example is Tead1 and Tead2 in notochord development [92]. In case of mES cells, three members of Klf family genes such as Klf2, Klf4, and Klf5 are expressed in undifferentiated state. Only simultaneous knockdown of these three (not any of the combination) shows the collapse of the network [93]. In addition to the compensation by the family gene, there is a genetic interaction between genes. To understand these complexities, it is necessary to establish compound KO cells. Recently, a computational modeling approach revealed that combination of gene knockdown can be predicted and some are sufficient to induce the network collapse in ES cells [94].

## Is this capturing in vivo pluripotency in vitro?

### LIF‐Stat3 signaling pathway

The question comes from the developmental biological aspect: LIF signaling plays an important role in culturing ES cells in vitro, but how does this signal work in vivo? LIF is expressed in the blastocyst‐stage embryo only in trophectoderm cells, and its receptor Lifr and gp130 are expressed in the ICM, detected by mRNA in situ hybridization [95]. Although it is expressed, it was unclear if this signal is active in this stage of embryo. Immunostaining of phospho‐Stat3 suggests that at least the signal is active in ICM cells [96], but *Stat3* KO embryos survive over implantation, and die around E6‐7 [97]. *Lif*, *Lifr*, and *gp130* KO embryos are known to develop normally until around mid‐gestation. *Lif* KO mice are born, but with female infertility because blastocysts are unable to implant in *Lif* (‐/‐) uteri [98]. *Lifr* KO mice have a perinatal lethality with a defect in motor neuron development [99, 100]. *gp130* KO mice die around E12.5‐16.5 due to cardiac and hematopoietic defects [101]. From these, the LIF signal was thought not to be essential for the establishment of pluripotent epiblast cells.

Embryonic diapause is a phenomenon observed in some mammals to keep the embryo un‐implanted in the uterus during lactation [102]. Interestingly, ES cells were first established from embryos in diapause [21]. New insight came after the analysis of *Lifr* and *gp130* mutant embryos in a diapause [103]. *Lifr* and *gp130* compound heterozygotes were intercrossed to generate double‐mutant embryos, and pregnant mothers were ovariectomized to induce diapause. After a certain period of diapause, embryos lacking both gp130 and Lifr transferred into pseudopregnant females could not be recovered. *gp130* KO embryos in diapause showed no surviving epiblast cells and culture outgrowth from *gp130* (‐/‐) embryo yielded only extraembryonic endoderm cells. Recently, maternal/zygotic *Stat3* null embryos have been analyzed, and revealed its requirement for the maintenance of pluripotent ICM cells during implantation [96]. These results strongly support the idea that Stat3 activation by LIF or other stimuli is required to keep the pluripotent ICM in vivo as well as in vitro.

### Two kinases inhibition in vivo

How about the 2i cultured cells? 2i medium consists of inhibitors of two protein kinase: Mek and GSK3. ES cells can be propagated without LIF in this condition. Mek is a component of FGF‐Mapk signaling and GSK3 is a component of the beta‐catenin destruction complex, which is involved in the canonical Wnt signaling pathway. Here I try to uncover one by one.

### Mek inhibition

Among the 22 Fgf ligands and four receptors [104], only Fgf4 and Fgfr2 disruption resulted in pre‐implantation lethality [71, 105]. As described above, Fgf4 is produced by undifferentiated ES cells, but is not required for either the maintenance or growth of pluripotent cells in vitro [72]. Rather it is required for differentiation [73]. How does this signal work in the embryo? Single‐cell gene expression analysis at E3.5 revealed that *Fgf4* is expressed in the *Nanog*‐positive epiblast lineage and its receptor *Fgfr2* is expressed in *Gata6*‐positive primitive endoderm cells [106]. The effect of Fgf inhibition during pre‐implantation development was examined by adding these inhibitors in the culture medium from different time points of development [107]. When eight‐cell‐stage embryos are cultured with these inhibitors or Mek inhibitor alone for 2 days, the primitive endoderm lineage marked by the expression of Gata4 protein is almost completely blocked and every ICM cell expresses Nanog. Injecting these ICM cells into another blastocyst revealed that these cells possess pluripotency. However, if the two inhibitors are added after blastocyst formation, at E3.75 then the embryos cultured for 2 days, embryonic development, and lineage segregation are not affected.

Yamanaka et al. [1] performed the opposite experiment for analyzing Fgf function in primitive endoderm formation. When 2–4‐cell‐stage embryos (E1.5) were treated with a very high concentration of Fgf4 with heparin, this resulted in the conversion of ICM cells into Gata6‐positive primitive endoderm cells at the expense of Nanog‐positive epiblast lineage cells. From these observations, Mek inhibition suppresses PrEn and enhances lineage commitment of ICM cells towards epiblast lineage.

### GSK‐3 inhibition

GSK‐3 is a serine/threonine kinase that is widely expressed and consists of two different gene products, GSK‐3α and GSK‐3β [108, 109, 110]. GSK‐3α and GSK‐3β have a highly conserved kinase domain, so most of the inhibitors affect both of them together [111]. One of the functions of GSK‐3 is to interact with scaffolding protein Axin and Adenomatous polyposis coli (APC) to make β‐catenin destruction complex. This complex phosphorylates the N‐terminus of β‐catenin, and phosphorylated β‐catenin is then ubiquitinated, followed by degradation with proteasome. When Wnt ligands bind to its receptor Frizzled and Lrp5/6, GSK‐3 does not phosphorylate β‐catenin, resulting in escape from proteasome‐mediated degradation. This β‐catenin translocates into the nucleus and binds to Tcf3 [112]. Tcf3 belongs to the repression type of transcription factors, and β‐catenin binding abrogates the repressor function of Tcf3 to continue to express pluripotency‐associated genes. Consistent with this, *Tcf3* KO ES cells show pronounced delay to exit from pluripotency [113, 114]. KO embryos show delayed shut down of pluripotency genes during gastrulation, and inhibit mesodermal gene expression [115].

Recombinant Wnt3a added to culture media is reported to enhance the self‐renewing activity of mES cells [116, 117], but the importance of the Wnt signal in the in vivo blastocyst‐stage embryo is not well characterized because there is no evidence for Wnt function in blastocyst‐stage embryo by KO studies. Axin2 is the direct target of canonical Wnt signaling pathway, so this gene is used as a marker to visualize active Wnt signals [118]. Pre‐implantation‐stage embryos express the *Axin2*‐LacZ reporter only in the ICM regions and its expression diminishes after implantation [119]. The Wnt signal might be active in the developing and proliferating naïve pluripotent cell, but this signal itself is not necessary for establishment and maintenance of the pluripotent state in vivo, shown by *Porcn* KO embryo [120]. From these observations, it is unclear that the Wnt signal plays a role in enhancing the pluripotent state in vivo, and it is necessary to analyze these KO embryos in diapause to conclude in future.

### Gene expression comparison between ES cells and ICM cells

Recent technology revealed the gene expression profile from small amounts of cells, and even from a single cell [106, 121] of peri‐implantation‐stage embryo. Tang et al. compared gene expression profiles during the process of ICM outgrowth to ES cells. They showed the difference between E3.5 ICM and ES cells cultured in serum and LIF. Most recently, Boroviak et al. [122] examined the gene expression profile from the pre‐implantation‐stage to post‐implantation‐stage epiblast and compared gene expression with cells cultured in 2i or 2i and LIF. According to the gene expression profile by Boroviak et al., naïve ES cells are most similar to the epiblast cells at E4.5 after primitive endoderm cells are segregated. They also showed efficient establishment of ES cell lines from E4.5 epiblast.

### Is EpiSC relevant to post‐implantation epiblast?

EpiSC are stem cells that can be established from a broad range of developmental stages from E5.5 to E8.0. It is important to culture the cells expressing Oct3/4 to establish the stem cell line [123]. Although their origins vary, the cells in culture have very similar gene expression profiles, and the expression of lineage marker genes, for example T or Sox17, are heterogeneous. Global gene expression pattern analysis shows that EpiSC lines in culture are most similar to the anterior primitive streak cells in the gastrulation‐stage embryo [124]. Basic features of EpiSC are conserved between lines, but they are variable between labs because the culture condition is slightly different. Essential components to support their self‐renewal are activin and Fgf signal. Some labs used feeder cells and bFgf only relying on activin production by feeders and endogenous Nodal, but others used serum or KSR in addition to activin A and bFgf. Recent findings by two independent groups suggest the model by which we can maintain the EpiS cell in a more pure state. They used the addition of Wnt signal inhibitor XAV939 [125, 126]. This is a reasonable component because Wnt signal in the post‐implantation embryo enhances mesoderm and endoderm formation and heterogeneity observed in the culture are spontaneous differentiation of T or Sox17‐positive cells.

## Conclusions

Since the establishment of mES cells as a genuine pluripotent stem cell, the secrets behind it have been uncovered one by one. Especially, after the discovery of LIF as an essential external signal, the analysis of the gene regulatory network downstream of this signal is intensively progressed and key transcription factors have been identified. After the discovery of 2i culture, we can keep the mES cells in the homogeneous manner as a ground state. This means we have been able to overcome the differences potentially existing in the different laboratories to further investigate properties of the naïve state in vitro. Gene expression analysis by Boroviak et al. [122] supports the idea that this ground state is not an artifact but rather it is captured in vivo pluripotent cell in vitro.

On the other hand, we have not found a core gene regulatory network of primed cells yet. EpiSCs can be a very good tool to analyze different states of pluripotency, which might be governed by different sets of pluripotency gene regulatory networks with lineage marker genes like Otx2 [127, 128] or Eomesodermin [129]. It is unclear why these cell lines lose germ line competency [42]. Interestingly, except for mice and rats, it is very difficult to establish naïve pluripotent stem cells in other mammals. The developing human embryo has a slightly different morphological feature compared with mice at the late blastocyst stage. In human, ICM cells at late blastocyst stage make a single layer of epiblast cells overlying the extraembryonic layer before implantation, on the other hand, the mouse epiblast at this stage is a three‐dimensional aggregate under the extraembryonic endoderm layer. This evokes the speculation that the human embryonic stem cell is more natural and stable in the primed than naïve state, which might exist only at the early blastocyst stage in a small time window in human. In addition to this thought (i.e., naïve or primed), so far, it seems to be very difficult to establish non‐human primate chimeric animals by injecting cultured pluripotent stem cells. The reason for this difficulty is not clear yet, however the fact that EpiSC can make chimeras only when injected into post‐implantation epiblast suggests that we need to capture the proper naïve state from those animals.

Compared to human, in mice, we now understand well what kind of cell identity it has and how similar mES cells are to their in vivo counterparts, so it is time to analyze how to start to differentiate. We know ES cells can differentiate, but little is known how. Recently, some reports tried to identify new molecules that are required for the exit from the ground state [130, 131, 132]. Mouse ES cells have a longer history than human, however knowledge about human ES cell are rapidly accumulated. Recently, human ES cells that have a similar gene expression signature to mouse naïve state have been reported [133, 134]. From these reports, we are starting to open the gate for proper human naïve cells. In conclusion, I optimistically speculate in the near future that we can manage to capture and control the undifferentiated state and differentiation of various types of pluripotent stem cells (which might be reflecting different stages of embryonic development) from various animals in vitro.

## Acknowledgments

I thank Austin Smith and Jenny Nichols for critical reading of this manuscript and helpful comments and discussions. I also thank Joerg Betschinger for the initial discussion about this manuscript. The author works in the laboratory of Austin Smith, funded by the Wellcome Trust and Medical Research Council.

### Conflict of interest

Masaki Kinoshita declares that he has no conflicts of interest to disclose.
